# Nicotinamide N-methyltransferase enhances resistance to 5-fluorouracil in colorectal cancer cells through inhibition of the ASK1-p38 MAPK pathway

**DOI:** 10.18632/oncotarget.9962

**Published:** 2016-06-13

**Authors:** Xinyou Xie, Huixing Liu, Yanzhong Wang, Yanwen Zhou, Haitao Yu, Guiling Li, Zhi Ruan, Fengying Li, Xiuhong Wang, Jun Zhang

**Affiliations:** ^1^ Department of Clinical Laboratory, Sir Run Run Shaw Hospital, School of Medicine, Zhejiang University, Hangzhou, Zhejiang 310016, P.R. China; ^2^ Key Laboratory of Biotherapy of Zhejiang Province, Hangzhou, Zhejiang 310016, P.R. China

**Keywords:** nicotinamide N-methyltransferase, 1-methylnicotinamide, 5-fluorouracil, colorectal cancer, p38 MAPK

## Abstract

Nicotinamide N-methyltransferase (NNMT), which converts nicotinamide to 1-methylnicotinamide (1-MNA), is overexpressed in a variety of human cancers and serves as a potential anti-cancer target. In this study, we investigated the effect of NNMT on 5-fluorouracil (5-FU) sensitivity of colorectal cancer (CRC) cells, and the underlying mechanisms. Our results show that down-regulation of NNMT in CRC HT-29 cells diminishes 5-FU resistance, while over expression of NNMT in SW480 cells enhances it. NNMT reduces reactive oxygen species (ROS) production induced by 5-FU by increasing 1-MNA in CRC cells. The reduction in ROS leads to inactivation of the ASK1-p38 mitogen-activated protein kinase (MAPK) pathway, which reduces 5-FU-induced apoptosis. *In vivo*, NNMT attenuates 5-FU-induced inhibition of CRC tumor growth in nude mice. These observations suggest that NNMT and the 1-MNA it produces inhibit the ASK1-p38 MAPK pathway, resulting in increased CRC cell resistance to 5-FU.

## INTRODUCTION

Colorectal cancer is one of the most commonly diagnosed cancers in the world, with over 1.3 million new cases diagnosed each year [1]. Chemotherapy is still an important adjuvant treatment for CRC, although surgical resection has been the primary treatment modality. 5-FU is widely used in the treatment of various cancers and is the standard first-line chemotherapeutic drug for CRC. However, patients who initially respond to 5-FU ultimately develop resistance; this has been a major obstacle in advanced CRC chemotherapy [2]. Therefore, there is an urgent need to understand the mechanisms responsible for the 5-FU resistance in CRC cells.

Resistance to 5-FU may occur by a number of mechanisms, including changes in metabolism, drug action, or signaling pathways that regulate apoptosis and autophagy in response to DNA damage [3]. The p38 MAPK signaling pathway has been shown to be a major mediator of 5-FU resistance in CRC cells [4].

NNMT, an S-adenosylmethionine-dependent enzyme with a molecular mass of 29 kDa, catalyzes N-methylation of nicotinamide, pyridines and other structural analogs. NNMT is over-expressed in various tumors, including glioblastoma [5], pancreatic cancer [6], papillary thyroid cancer [7], renal carcinoma [8], colorectal cancer [9, 10], oral squamous cell carcinoma [11], lung cancer [12], and bladder cancer [13]. NNMT has been considered as a novel and sensitive serum biomarker for the detection of CRC [9]. Moreover, NNMT has been associated with radiation resistance in bladder cancer cells [13] and cancer stem cells [14].

Our recent studies have shown that NNMT enhances tumorigenesis in human CRC cells by inhibiting apoptosis and promoting cell cycle progression, and that the cellular effect of NNMT is mediated by the produced 1-methylnicotinamide (1-MNA) [15]. Our results have suggested that NNMT is involved in energy balance and ROS production, and is associated with PI3K/Akt and MAPK pathways activation.

In this study, we investigated the effect of NNMT expression on the 5-FU sensitivity of CRC cells. We demonstrate that NNMT inhibits activation of the ASK1-p38 MAPK pathway via 1-MNA production. Inactivation of ASK1 decreases apoptosis via p38 MAPK pathway to enhance 5-FU resistance in CRC cells.

## RESULTS

### NNMT reduces sensitivity to 5-FU in CRC cells

Human CRC cells SW480 lack constitutive NNMT expression, while HT-29 cells have high levels of endogenous NNMT expression. SW480 cells transfected with either pcDNA3.1-NNMT vector (SW480/NNMT-1, SW480/NNMT-2) or pcDNA3.1 control vector (SW480/Vector), and HT-29 cells infected with lentiviral shRNA-NNMT (HT-29/NNMT shRNA 1#, HT-29/NNMT shRNA 2#) or lentiviral shRNA NC as a negative control (HT-29/NC), were successfully constructed (Supplementary Figure S1).

**Figure 1:**
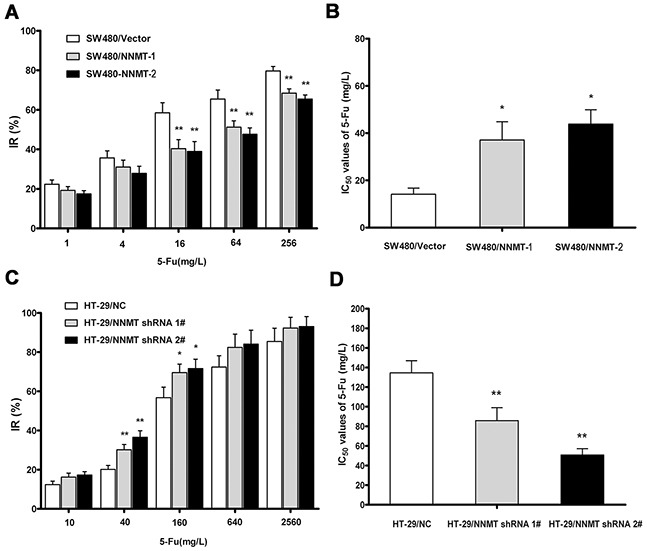
NNMT reduces sensitivity to 5-FU in CRC cells **A, C.** The cells were exposed to various concentrations of 5-FU for 48 h, and the IRs were assessed by MTT assay. Data are presented as mean ± SD (n = 5). **B, D.** 5-FU resistance was evaluated by IC_50_. Data are presented as mean ± SD (n = 5) (* *p* < 0.05, ** *p* < 0.01).

To investigate whether NNMT expression affects the cellular sensitivity to 5-FU, the 5-FU sensitivity of cells treated with 5-FU or DMSO for 48 h was evaluated as IC_50_ by MTT assay. The inhibition rates (IR) in SW480/NNMT-1 and SW480/NNMT-2 cells were lower than in SW480/Vector cells at various concentration of 5-FU (Figure 1A). The IC_50_ values of 5-FU in SW480/NNMT-1 (37.08 ± 7.74 mg/L) and SW480/NNMT-2 (43.85 ± 6.04 mg/L) cells were significantly higher than in SW480/Vector cells (14.13 ± 2.60 mg/L) (Figure 1B). The IR of HT-29/NNMT shRNA 1#, and HT-29/NNMT shRNA 2# cells were higher than HT-29/NC cells after treatment with various concentrations of 5-FU (Figure 1C). The IC_50_ values of 5-FU in HT-29/NNMT shRNA 1# (85.83 ± 13.20 mg/L) and HT-29/NNMT shRNA 2# cells (50.79 ± 6.35 mg/L) were lower than in HT-29/NC cells (134.56 ± 12.39 mg/L) (Figure 1D). These results indicate that NNMT expression reduces cellular sensitivity to 5-FU.

### NNMT reduces apoptosis *induced by 5-FU in CRC cells*

**Figure 2:**
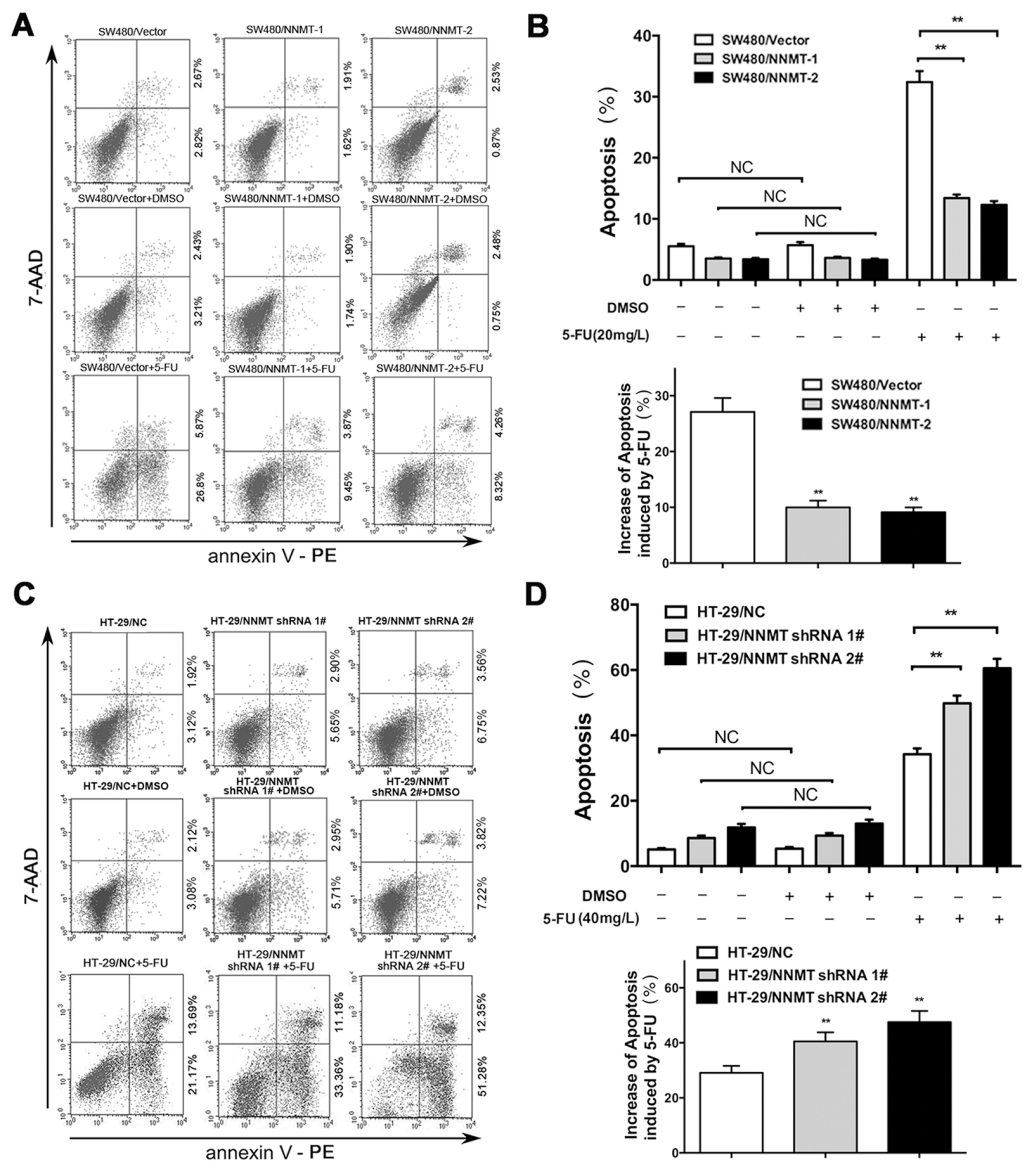
NNMT reduces apoptosis in 5-FU induced CRC cells **A, C.** The cells were treated for 48 h with the indicated dose of 5-FU or vehicle (DMSO). Apoptosis was evaluated by Annexin V-PE and 7-AAD after 5-FU treatment. **B, D.** Histogram shows the combination of the results of three independent experiments performed as in (A) and (C). Data are presented as mean ± SD (** *p* < 0.01) (n = 3).

To explore the mechanisms involved in 5-FU resistance induced by NNMT, cell apoptosis was examined by flow cytometry (FCM). There was no significant difference in apoptosis between cells treated with DMSO (vehicle for 5-FU) and the group without DMSO. After treatment with 5-FU (20 mg/L for SW480 cells, and 40 mg/L for HT-29 cells) for 48 h, a much lower percentage of apoptosis was observed in SW480/NNMT-1 (13.42 ± 1.04%) and SW480/NNMT-2 cells (12.39 ± 1.18%), compared to SW480/Vector cells (32.38 ± 3.06%) (Figure 2A, 2B). In contrast, down-regulated NNMT expression in HT-29 cells enhanced the 5-FU-induced apoptosis. The percentages of apoptotic cells in HT-29/NNMT shRNA 1# (49.45 ± 3.67%) and HT-29/NNMT shRNA 2# (62.54 ± 3.12%) were significantly higher than in HT-29/NC (33.45 ± 2.50%) (Figure 2C, 2D). By comparing the apoptosis of cells only treated with DMSO, the results showed that overexpression of NNMT in SW480 cells significantly reduced the increase of apoptosis induced by 5-FU, which was enhanced by down-regulation of NNMT in HT-29 cells.

To further confirm the effect of NNMT on 5-FU induced apoptosis in CRC cells, activation of apoptosis-related proteins, caspase-3, caspase-8, and caspase-9, was analyzed by western blotting. Compared with HT-29/NC cells, HT-29/NNMT shRNA 1# and shRNA 2# cells treated with 5-FU showed activation of cleaved caspase-3, -8 and -9. The opposite results were found in SW480/Vector, SW480/NNMT-1 and SW480/NNMT-2 cells. Overexpression of NNMT downregulated cleaved caspase-3, -8 and -9 (Supplementary Figure S2). These results indicate that NNMT expression reduces the 5-FU induced apoptosis in CRC cells.

### NNMT inhibits activation of p38 MAPK in 5-FU-treated CRC cells

**Figure 3:**
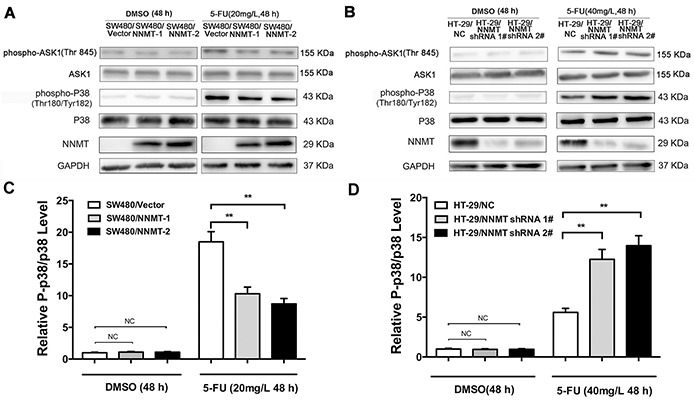
NNMT inhibits the activation of ASK1-p38 MAPK pathway in 5-FU-induced CRC cells Cells were treated for 48 h with the indicated dose of 5-FU or vehicle (DMSO). **A, B.** The levels of ASK1, p-ASK1, p38 and p-p38 were analyzed by Western blot. The relative P-p38/p38 levels after protein quantification of the western blot results were shown in **C.** and **D.** compared to the control group, which was normalized as 1, respectively. GAPDH was used as internal control. The data are representative of three experiments.

**Figure 4:**
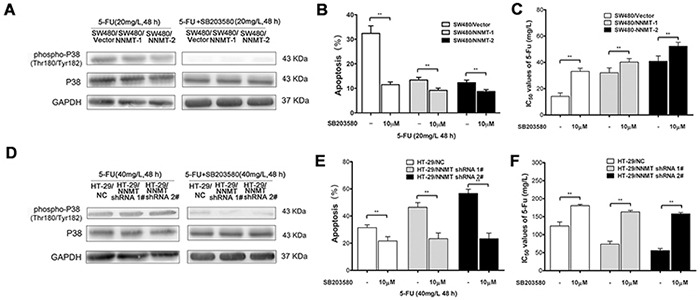
The inhibition of p38 MAPK pathway affects NNMT-related 5-FU resistance in SW480 and HT-29 cells **A, D.** Cells were treated with 5-FU after pre-treatment with 10 μM of SB203580 for 48 h. The phosphorylation levels of p38 were examined by Western blot. GAPDH was used as internal control. The data are representative of three experiments. **B, E.** Histogram shows the combination of the apoptosis results of three independent experiments. Data are presented as mean ± SD (** *p* < 0.01). **C, F.** 5-FU resistance was evaluated by IC_50_. Data are presented as mean ± SD (n = 5) (* *p* < 0.05, ** *p* < 0.01).

To further explore the potential mechanism by which expression of NNMT inhibits the 5-FU-induced apoptosis, we examined the involvement of p38 MAPK. Phosphorylation levels of p38 were very low in cells treated only with DMSO. After treatment with 5-FU, p38 phosphorylation sharply increased. The phosphorylation levels of p38 were significantly lower in SW480/NNMT-1 and SW480/NNMT-2 cells compared with SW480/Vector cells after 5-FU treatment (Figure 3A). In contrast, the phosphorylation levels of p38 were significantly higher in HT-29/NNMT shRNA 1# and HT-29/NNMT shRNA 2# cells compared with HT-29/NC cells (Figure 3B). The relative P-p38/p38 levels showed the same trend (Figure 3C, 3D). To determine the role of p38 in NNMT-mediated 5-FU resistance, 5-FU-treated CRC cells were incubated with SB203580, a specific p38 inhibitor. There was no significant difference in apoptosis in cells treated only with (or without) SB203580 (10 μM) (Supplementary Figure S3). When the phosphorylation levels of p38 was inhibited by SB203580 (10 μM) after incubation with 5-FU for 48 h, apoptosis decreased in all cells, and did not significantly differ between SW480/NNMT-1, SW480/NNMT-2 and SW480/Vector cells, and between HT-29/NNMT shRNA 1#, HT-29/NNMT shRNA 2# and HT-29/NC cells (Figure 4). Along with the change in apoptosis, the IC_50_ value of 5-FU markedly increased in all cells, and did not significantly differ between SW480/NNMT-1, SW480/NNMT-2 and SW480/Vector cells, and between HT-29/NNMT shRNA 1#, HT-29/NNMT shRNA 2# and HT-29/NC cells (Figure 4C, 4F). These results indicate that p38 MAPK is an important mediator of apoptosis in NNMT-induced 5-FU resistance in CRC cells.

### NNMT inhibits activation of ASK1 by reducing intracellular ROS levels in 5-FU-treated CRC cells

To evaluate how NNMT expression inhibits the activation of p38 MAPK, we next examined the activation of ASK1, an upstream signal of p38. To measure ASK1 activation, we analyzed the phosphorylation levels of ASK1 Thr^845^. After treatment with 5-FU for 48 h, the phosphorylation levels of ASK1 in all groups sharply increased. A lower phosphorylation level of ASK1 was observed in SW480/NNMT-1 and SW480/NNMT-2 cells after incubation with 5-FU, whereas a higher phosphorylation level of ASK1 was found in SW480/Vector cells (Figure 3A). In contrast, down-regulated NNMT expression in HT-29 cells increased p-ASK1 Thr^845^ levels after 5-FU treatment (Figure 3B). Together, these results indicate that NNMT expression decreases apoptosis through inactivating the ASK1-p38 pathway in 5-FU treated CRC cells.

**Figure 5:**
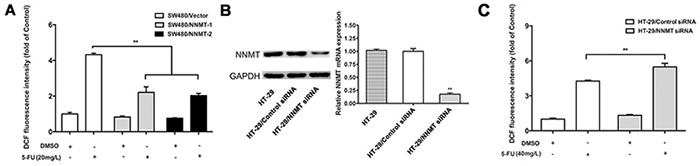
NNMT reduces the ROS production in CRC cells after treatment with 5-FU The intracellular ROS was detected after 48 h by flow cytometry using the fluorescent probe 2′,7′-dichlorodihydrofluorescein diacetate. Results are represented as fold of DCF fluorescence compared to control. ROS decreased in SW480 cells treated with pcDNA3.1/NNMT **A.** and increased in HT-29 cells treated with NNMT siRNA **C, B.** Expression of NNMT in HT-29 cells transfected by siRNAs at a final concentration of 80 nM was analyzed by real-time RT-PCR and western blot. GAPDH was used as an internal control. The western blot is representative of at least three independent experiments. The mRNA levels were normalized to GAPDH levels and all values are shown compared to the control values, which were assumed to be 1. There was a statistical significance between cells transfected with NNMT siRNA and control siRNA (**p < 0.01). Values are expressed as means ± SD of three independent experiments. (** *p* < 0.01 vs. control group).

We have previously shown that NNMT reduces the intracellular ROS levels in CRC cells [15]. To explore the potential mechanism responsible for the AKS1 inactivation by NNMT in 5-FU treated CRC cells, we have analyzed the intracellular ROS, which can activate ASK1 by dissociating it from glutathione-S-transferase (GST). The levels of ROS in each group sharply increased after treatment with 5-FU for 48 h. Up-regulation of NNMT significantly reduced the ROS production in 5-FU treated SW480 cells, whereas down-regulation of NNMT significantly enhanced the ROS production in 5-FU-treated HT-29 cells (Figure 5).

These results indicate that NNMT inhibits activation of the ASK1-p38 pathway to prevent apoptosis through reducing ROS production in 5-FU teated CRC cells.

### 1-MNA inhibits intracellular ROS levels and ASK1-p38 MAPK pathway, resulting in decreased *apoptosis* in 5-FU-treated CRC cells

**Figure 6:**
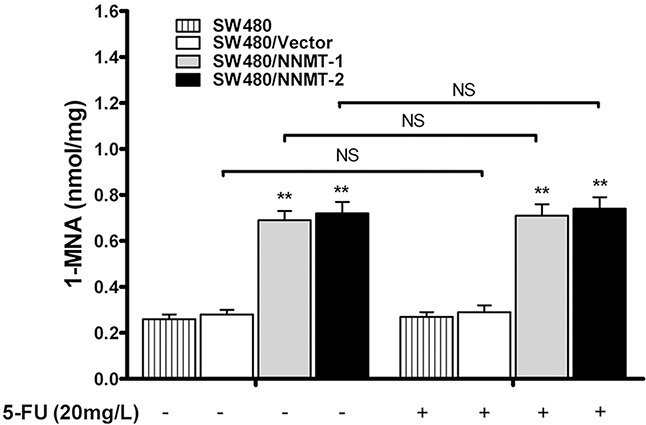
NNMT increases intracellular level of 1-MNA in CRC cells independently of 5-FU The intracellular 1-MNA was detected after 48 h by HPLC-UV. Values are expressed as means ± SD of three independent experiments (** *p* < 0.01 vs. control group).

**Figure 7:**
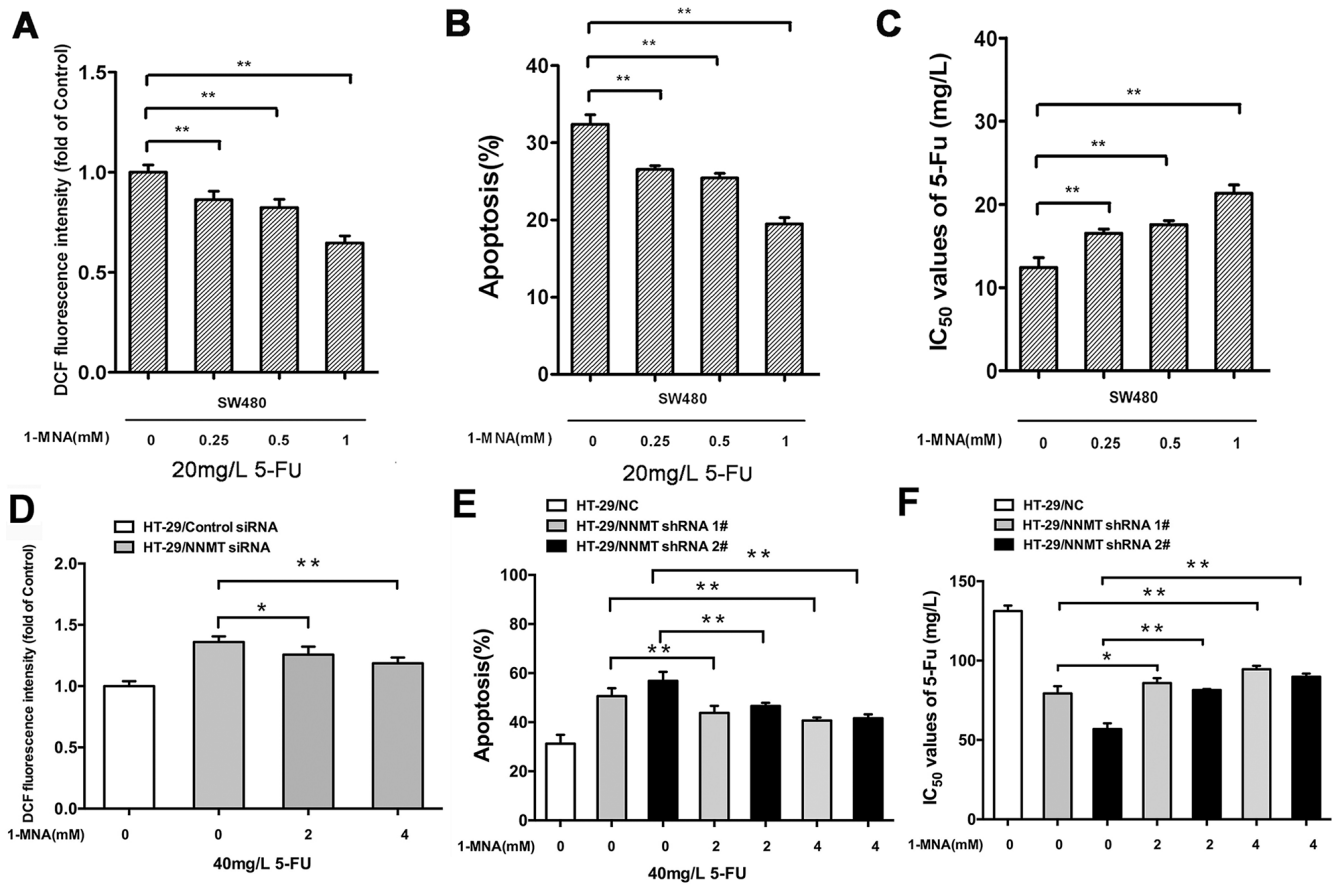
1-MNA attenuates ROS production and inhibits apoptosis after treatment with 5-FU to enhance resistance in CRC cells **A, D.** Cells were incubated with increasing concentration of 1-MNA and 5-FU for 48 h, after which the intracellular ROS was measured by flow cytometry. Results are represented as fold of DCF fluorescence compared to the group without 1-MNA. **B, E.** Histogram shows combination of the apoptosis results of three independent experiments. **C, F.** 5-FU resistance was evaluated by IC_50_. Values are expressed as means ± SD of three independent experiments (NS= not significant; * *p* < 0.05 vs. control group; ** *p* < 0.01 vs. control group).

We have previously shown that NNMT decreases the intracellular levels of ROS via 1-MNA, the metabolic product of NNMT [15]. To investigate the function of NNMT in 5-FU-treated CRC cells, we analyzed the intracellular 1-MNA levels. Overexpression of NNMT significantly increased 1-MNA in SW480/NNMT-1 and SW480/NNMT-2 cells with or without 5-FU treatment compared with SW480/Vector cells (Figure 6). The cellular levels of 1-MNA exhibited no significant changes between groups with 5-FU and without 5-FU, suggesting that NNMT increases the 1-MNA production independently of 5 -FU. To explore the 1-MNA role in 5-FU resistance in CRC cells, SW480 cells were incubated with increasing concentrations of 1-MNA (0.25 mM, 0.5 mM and 1 mM) instead of overexpressing NNMT. First, SW480 cells treated with increasing concentrations of 1-MNA showed markedly decreased intracellular ROS levels compared with cells without 1-MNA (Figure 7A). Second, a marked decrease in apoptosis was observed in SW480 cells treated with 1-MNA, compared with cells without 1-MNA (Figure 7B). Third, the IC_50_ value of 5-FU was increased through the decrease of apoptosis in SW480 cells with increasing concentrations of 1-MNA (Figure 7C). In the rescue experiment of 5-FU-treated HT-29 cells, the intracellular ROS level and apoptosis of HT-29/NNMT shRNA 1# and HT-29/NNMT shRNA 2# decreased by incubation with 1-MNA (2 mM and 4 mM) (Figure 7D, 7E). The IC_50_ of 5-FU increased through the decrease of apoptosis (Figure 7F), indicating that 1-MNA can partly rescue the phenotype lacking NNMT. These results indicate that NNMT enhances resistance to 5-FU by decreasing 1-MNA-mediated apoptosis of CRC cells.

**Figure 8:**
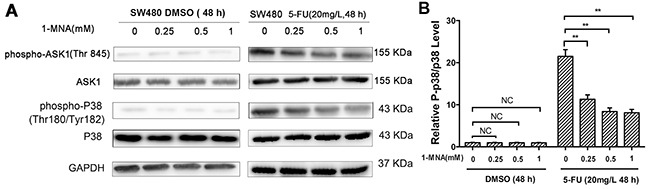
1-MNA attenuates activation of p38 MAPK pathway in SW480 cells after treatment with 5-FU **A.** SW480 cells were incubated with increasing concentration of 1-MNA (0, 0.25, 0.5, 1 mM) and 5-FU (20 mg/L) or vehicle (DMSO) for 48 h, after which the levels of ASK1, p-ASK1, p38 and p-p38 were analyzed by Western blot. GAPDH was used as internal control. The data are representative of three experiments. **B.** The ratio of p-p38/p38 after protein quantification of the western blot results was shown in (A) compared to the group treated with only DMSO, which was normalized as 1.

Our results indicate that 1-MNA has no significant effect on the ASK1-p38 MAPK pathway. However, activation of ASK1 and p38 was decreased in SW480 cells treated with 1-MNA after exposure to 5-FU (Figure 8).

### NNMT attenuates 5-FU suppression of tumor growth via 1-MNA *in vivo*

**Figure 9:**
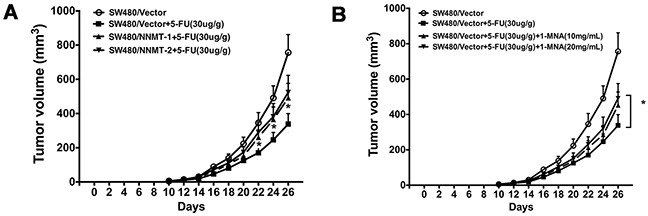
NNMT attenuates 5-FU suppression of tumor growth via 1-MNA *in vivo* **A.** SW480/Vector, SW480/NNMT-1 and SW480/NNMT-2 cells (3×10^6^) were subcutaneously injected into the upper portion of the right hind limb of nude mice (n=6 for each group). Treatment was started on the tenth day after injection. 5-fluorouracil (30 mg/kg) or PBS (for control group) were administered via intraperitoneal injection every 2 days. **B.** The two groups of SW480/Vector were also fed 1-MNA in the water (10 or 20 mg/mL) in the four SW480/Vector groups. The group treated with only 5-FU is the control group. Values are expressed as means ± SD. (*p* < 0.05).

**Figure 10:**
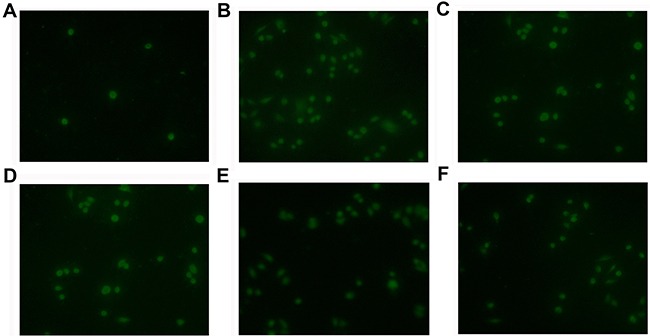
NNMT attenuates the 5-FU-induced apoptosis via 1-MNA *in vivo* Apoptosis of tumor cells treated with 5-FU (30 mg/kg) and 1-MNA was detected by TUNEL analysis. **A.** SW480/Vector cells, **B.** SW480/Vector cells treated with 5-FU, **C.** SW480/NNMT-1 cells treated with 5-FU, **D.** SW480/NNMT-2 cells treated with 5-FU, **E.** SW480/Vector cells treated with 5-FU and 1-MNA (10 mg/mL), **F.** SW480/Vector cells treated with 5-FU and 1-MNA (20 mg/mL). The cells overexpressing NNMT or treated with 1-MNA showed less cell death than SW480/Vector cells (*p* < 0.05). Original magnification: ×400.

Our *in vitro* data have shown that NNMT enhances resistance of CRC cells to 5-FU by decreasing 1-MNA-mediated apoptosis. To confirm these data *in vivo*, we used nude mice implanted with colorectal cancer xenografts. After treatment with 5-FU for 16 days, the tumors in mice implanted with SW480/NNMT-1 (251.67 ± 45.3 mm^3^) or SW480/NNMT-2 (273.89 ± 49.5 mm^3^) cells were significantly bigger compared to the control SW480/Vector group (158.45 ± 31.2 mm^3^) (Figure 9A). In addition, mice implanted with the control SW480/Vector cells, treated with 5-FU, and fed with 1-MNA had bigger tumor volumes compared to mice fed with water (Figure 9B). The TUNEL analysis of tumor cells showed that mice overexpressing NNMT or treated with 1-MNA exhibited less cell death induced by 5-FU than SW480/Vector cells (Figure 10). Consistent with the in vitro results, these data indicate that NNMT attenuates the 5-FU suppression of tumor growth via 1-MNA.

## DISCUSSION

NNMT is increased in CRC tumor tissues and serum of CRC patients [9, 10]. Our recent study has shown that NNMT overexpression increases cell survival, proliferation, colony formation, cell cycle progression, and intracellular ATP levels in CRC cells, and promotes tumorigenicity in mice [15]. Since suppression of NNMT inhibits proliferation of several types of human carcinoma cells [15–19], NNMT may become a possible molecular target for anti-cancer therapy.

Although 5-FU has been used as the first-line chemotherapy for CRC, the drug resistance has been a major obstacle [2]. Elucidating mechanisms responsible for the 5-FU resistance in CRC cells should improve effectiveness of 5-FU-based chemotherapy and reduce side effects. Loss of apoptotic control, which contributes to the survival of tumor cells, is one of the main causes of resistance to anticancer agents. We have previously found that NNMT, and its metabolic product 1-MNA, decrease intracellular ROS, resulting in decreased apoptosis [15]. Even though the relationship between 5-FU resistance and NNMT expression has not been explored, it is reasonable to speculate that NNMT is involved in the resistance to 5-FU in tumor cells. However, the role of NNMT in the 5-FU induced apoptosis is unknown. In this study, we investigated the NNMT role in 5-FU sensitivity of CRC cells.

Our results demonstrate that NNMT overexpression reduces 5-FU sensitivity of CRC SW480 cells, while NNMT downregulation increases sensitivity of CRC HT-29 cells. Our data show that NNMT overexpression reduces 5-FU-induced apoptosis in CRC cells, indicating that this may represent one of the mechanisms responsible for the 5-FU resistance. However, the specific pathway by which NNMT decreases apoptosis induced by 5-FU needs further studies.

One of the apoptotic signaling pathways is the ASK1-p38 MAPK, which has been identified as a transduction pathway of apoptotic signals initiated by outside stimuli, such as ROS. Study by de la Cruz-Morcillo et al showed that p38 MAPK activation is a key determinant in the cellular response to 5-FU, while ERK and JNK MAPK pathways are not involved [4]. They also demonstrated a critical role for the p38 MAPK in the cellular response to 5-FU by controlling the balance between apoptosis and autophagy via p53-driven apoptosis. In our study, we also found that 5-FU could activate p38 MAPK in CRC cells. Considering that NNMT inhibits activation of p38 MAPK to decrease apoptosis in 5-FU-treated CRC cells, we treated cells with SB203580 to inhibit the activation of p38 MAPK. When the phosphorylation levels of p38 were inhibited, apoptosis was markedly decreased in CRC cells. Together, these results indicate that p38 MAPK is an important mediator of apoptosis in NNMT-mediated 5-FU resistance.

The levels of 1-MNA in SW480/NNMT-1 and SW480/NNMT-2 cells treated with 5-FU were higher than in SW480/Vector cells, suggesting that NNMT inactivates the ASK1-p38 MAPK pathway through the 1-MNA production. Several activities of 1-MNA, the metabolic product of NNMT, have been described, such as anti-inflammatory activity in skin diseases, induction of prostacyclin synthesis via COX-2, aortal endothelium protection in diabetes and hypertriglyceridaemia, and increased survival rate of diabetic rats [20]. Our study suggests that 1-MNA might be a considerable compound in anti-cancer therapy.

In summary, this study provides the first demonstration that NNMT plays a role in the resistance to 5-FU in CRC cells. Our results indicate that NNMT and 1-MNA inhibit activation of the ASK1-p38 MAPK pathway to decrease apoptosis and enhance 5-FU resistance in CRC cells. Thus, NNMT has a novel function in 5-FU resistance, and might be a potential therapeutic target for enhancing the 5-FU pro-apoptotic effect in CRC treatment.

## MATERIALS AND METHODS

### Cell culture and reagents

The human colorectal cancer cell line SW480 and HT-29 (both from epithelial colorectal adenocarcinoma) were purchased from the American Type Culture Collection (Rockville, MD). The SW480/Vector, SW480/NNMT-1, SW480/NNMT-2 and HT-29/NC, HT-29/NNMT shRNA 1# and HT-29/NNMT shRNA 2 # cells were constructed by the method in our previous study [15]. SW480 cells were cultured in RPMI-1640 medium and HT-29 cells were cultured in McCoy's 5A medium. All media were supplemented with 10% fetal bovine serum, 100 U/mL of penicillin and 100 μg/mL of streptomycin, and cells were maintained at 37°C in a humidified 5% CO_2_ incubator.

The following antibodies were obtained from Cell Signaling Technology (Beverly, Massachusetts, USA): anti-ASK1, anti-phospho-ASK1 (Thr 845), anti-caspase-3, anti-cleaved caspase-3, anti-caspase-9, anti-cleaved caspase-9, anti-p38, anti-phospho-p38, anti-GAPDH. The mouse anti-NNMT monoclonal antibody 1E7 was prepared through the hybridoma technique as previously described [19]. Goat anti-mouse and goat anti-rabbit HRP-conjugated antibodies were obtained from Zhongshan Goldenbridge Biotechnology Co. (Beijing, China).

### Effect of NNMT on chemo-sensitivity to 5-FU

MTT assay was used to explore the effect of NNMT on chemo-sensitivity to 5-FU. The cells were prepared at a density of 4 × 10^4^/mL and 200μL aliquots were dispensed into 96-well flat-bottom plates. The cells were allowed to attach overnight and the medium was then removed and replaced with fresh medium containing 5-FU (Sigma, St. Louis, MO, USA), which was diluted in dimethyl sulfoxide (DMSO, Sigma) and stored at −20°C, with serial dilution at a final concentration of 0, 1, 4, 16, 64 and 256 mg/L for SW480 cells and 0, 10, 40, 160, 640 and 2560mg/L for HT-29 cells. After another 48 h incubation with 5-FU, 20 μL of the 5g/L MTT reagent (Sigma) was added to each well and cells were incubated for a further 4 h at 37°C. Subsequently, the supernatant was removed carefully and 150 μL of DMSO was added to each well. Finally, the absorbance value of each well was read at 490 nm using an ELISA plate reader instrument (Bio-Rad, Model 680, Japan) with DMSO as the blank. The inhibition rate (IR) was calculated by the equation: [1-(mean absorbance of drug wells/mean absorbance of control wells)] × 100%. Absorbance of each experimental well was adjusted by the mean absorbance of blank wells. 5-FU resistance was evaluated by IC_50_, which was determined as the concentration of the drug required when the IR was 50%.

### Apoptosis analysis

Apoptosis was detected by flow cytometric analysis using an Annexin V-PE/7-AAD Apoptosis Detection Kit (MultiSciences Biotech Co., Ltd., Hangzhou, China) according to the protocol provided. Briefly, the cells were seeded (3×10^5^ cells/well) in a six-well plate. After culturing for 48 h, the treated cells were harvested, incubated with Annexin V-PE and 7-AAD for 15 min at room temperature in the dark, and immediately analyzed by flow cytometry (FACSCalibur flow cytometer, BD, CA, USA). Each experiment was conducted at least three times.

### Western blot analysis

To evaluate levels of the related proteins (ASK1, p38, caspase-3, caspae-8, caspase-9, NNMT and GAPDH) cellular samples were analyzed by Western blot. Cell extracts were prepared with RIPA Lysis Buffer (Beyotime biotechnology, Haimen, China). Protein concentration was measured by the bicinchoninic acid (BCA) protein assay Kit (Beyotime biotechnology). A total of 40 μg of protein samples from each cell strain was subjected to 10 or 15% sodiumdodecyl sulfate-polyacrylamide gel electrophoresis (SDS-PAGE) and transferred to Immobilon P (Millipore, Billerica, MA, USA). After regular blocking and washing, the membranes were incubated with primary antibodies (1:1000 dilution) overnight at 4°C, followed by incubating appropriate secondary antibodies by standard procedures. Signals were visualized using enhanced chemiluminescence detection reagents (Millipore) and imaged using an Image Quant LAS-4000 (Fujifilm, Tokyo, Japan).

### Measurement of ROS production by flow cytometry

The measurement of intracellular ROS was performed by flow cytometry. Specific siRNAs (sc-61213, Santa Cruz Biotechnology, CA, USA) were chosen for silence NNMT expression to avoid the fluorescence of GFP in the lentiviral vector as previously described [15], which may interfere with the ROS measurement.

Briefly, cells were cultured in 60-mm dishes for 48 h and then treated with 20 μM 2′,7′-dichlorodihydrofluorescein diacetate (DCFH-DA, Sigma, St. Louis, MO, USA). The fluorescence intensity was measured to indicate the level of intracellular ROS at the provided time point with an emission at 530 nm and excitation at 485 nm by flow cytometry (FACSCalibur flow cytometer, BD, CA, USA). The results obtain for the cells treated with NNMT siRNA or pcDNA3.1/NNMT were compared to those obtained for the cells treated with control siRNA or vector, which were assumed to be one.

### HPLC-UV detection of 1-methylnicotinamide

The HPLC-UV method for detection of 1-MNA was described previously [15]. HPLC-UV was performed using a Hewlett-Packard 1100 photodiode array detector (Waldbronn, Germany) incorporating a 250×4.6-mm-inner-diameter Agilent TC-C18 5-μm reversed-phase column. The 100 μl mobile phase (20%, v/v methanol in solution A) with 20 nmol 1-MNA was monitored by absorbance at 265 nm which is identified to the peak corresponding to 1-MNA. A 0.06-11.6 nmol/100 μl 6-point standard curve for 1-MNA was produced, which was subsequently used to calculate the concentration of 1-MNA for all.

### Tumor growth *in vivo* and TUNEL assay

All of the animal experiments were previously approved by the Animal Care and Use Committee in the Sir Run Run Shaw Hospital of Zhejiang University (Permit Number: 20120222-31). The procedures were complied with the NIH Guide for the Care and Use of Laboratory Animals.

Male BALB/c nude mice (5 weeks of age, body weight of 16-18 g) were divided into six groups. 3×10^6^ cells of each of the SW480/Vector, SW480/NNMT-1 and SW480/NNMT-2 cell lines were subcutaneously injected into the upper portion of the right hind limb of nude mice (n=6 for each group). Treatment was started on the tenth day after injection. 5-fluorouracil (30 mg/kg) or PBS (for control group) were administrated via intraperitoneal injection every 2 days. Meanwhile, the two groups of SW480/Vector were also fed 1-MNA in the water (10 or 20 mg/mL). The tumor size was measured using calipers every two days and calculated using the following formula: *V* = π/6×A×B^2^, where V is the volume, A is the largest diameter, and B is the smallest diameter. With the exception of mice with large tumor burdens (A < 20 mm, B < 10 mm), the animals were killed through isoflurane inhalation (Abbot Laboratories Ltd., North Chicago, IL, USA) followed by cervical dislocation 26 days after injection. At the end of the experiment, the tumors were harvested and weighed.

Tumor tissues were fixed in 4% formaldehyde, dehydrated with gradient ethanol, and embedded in paraffin. Tissue sections (4μm) were then dewaxed and rehydrated according to a standard protocol. For TUNEL assay, an in situ apoptosis detection kit (Roche Diagnostics, Branchburg, NJ, USA) was used to detect apoptotic cells in tumor tissue sections. Briefly, after incubation with proteinase K and rinsing with ddH_2_O, endogenous peroxidase was blocked with 3% H_2_O_2_. Samples were then washed three times with PBS and incubated with the TUNEL reaction mixture, containing FITC-labeled dUTP and terminal deoxynucleotidyl transferase, for 1 h at 37°C in a humidified atmosphere in the dark. After incubation, the samples were washed three times with PBS and analyzed using the fluorescence microscopy system (Carl Zeiss, Thornwood, NY, USA). Use an excitation wavelength in the range of 488 nm and detection in the range of 550 nm. Three random fields were counted per slide in duplicate slides.

### Statistical analysis

All statistical analyses were carried out using the SPSS 19.0 statistical software package. All data were obtained from at least three individual experiments. Data are presented as mean ± SD. Statistical analysis between groups was performed by one-way ANOVA. IC_50_ values were calculated using regression analysis. P value < 0.05 was considered statistically significant.

## SUPPLEMENTARY FIGURES


