# Supplementary material for: Nicotinamide N-methyltransferase enhances resistance to 5-fluorouracil in colorectal cancer cells through inhibition of the ASK1-p38 MAPK pathway
Source: Oncotarget. 2016 Jun 13;7(29):45837–48. doi: 10.18632/oncotarget.9962 (PMC5216764; doi:10.18632/oncotarget.9962)
Supplement: Supplementary file 1 [file oncotarget-07-45837-s001.pdf]

Nicotinamide N-methyltransferase enhances resistance to 5-fluorouracil in colorectal cancer cells through inhibition of the ASK1-p38 MAPK pathway

SUPPLEMENTARY FIGURES

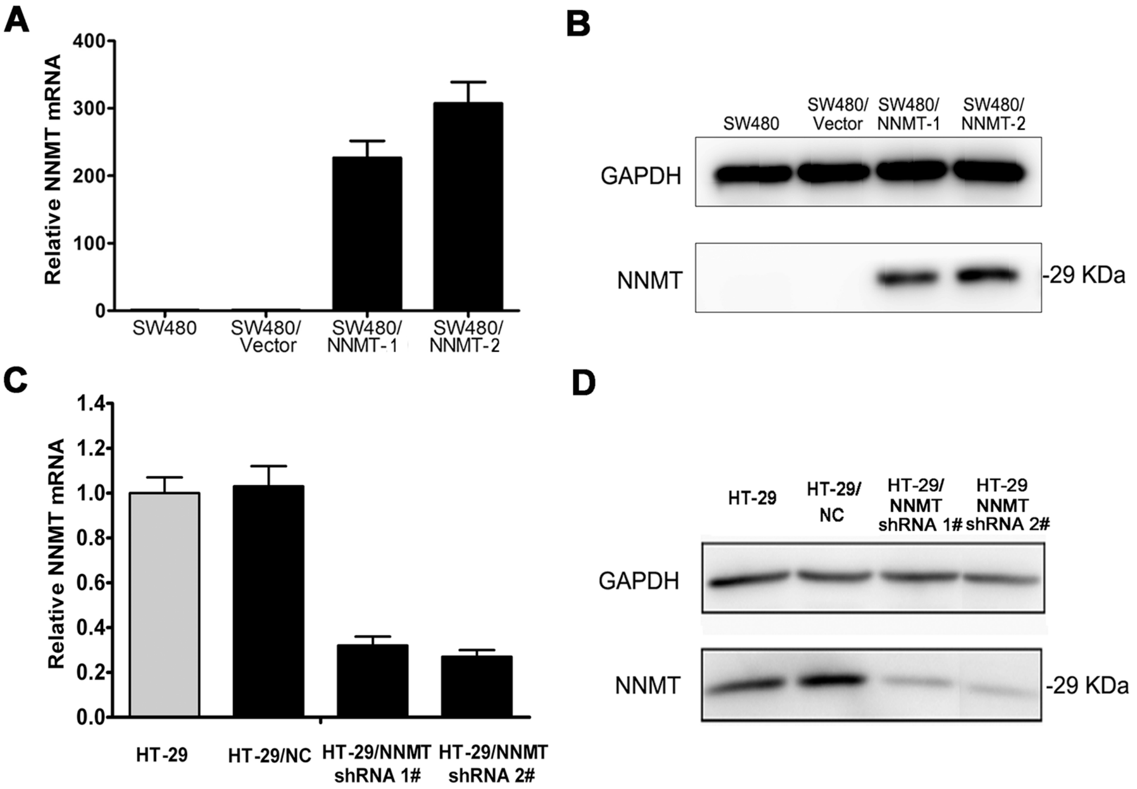

Supplementary Figure S1: Evaluation of NNMT expression in SW480, HT-29 and their cell models. A, C. Expression of NNMT mRNA was detected by Real-Time quantitative PCR. GAPDH was used as internal control. **B, D.** Expression of NNMT protein was detected by Western blot. GAPDH was used as internal control. The relative NNMT mRNA levels was determined using the 2(-ΔΔCt) method where ΔΔCt = ΔCt (treated groups) – ΔCt (Wild group) and ΔCt = (CtNNMT -CtGAPDH). Data are presented as Mean ± SD (n = 5).

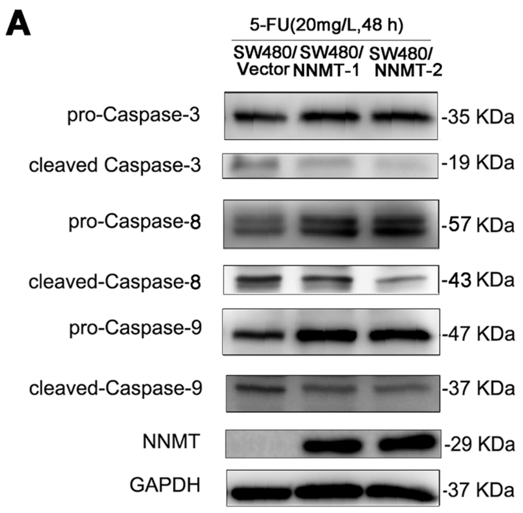

Supplementary Figure S2: NNMT decreases the activation of apoptosis-related proteins in 5-FU induced CRC cells. Cells were treated for 48 h with the indicated dose of 5-FU or vehicle (DMSO). The levels of caspase-3, caspase-8 and caspase-9, and their cleaved patterns were analyzed by Western blot. The data are representative of three experiments.

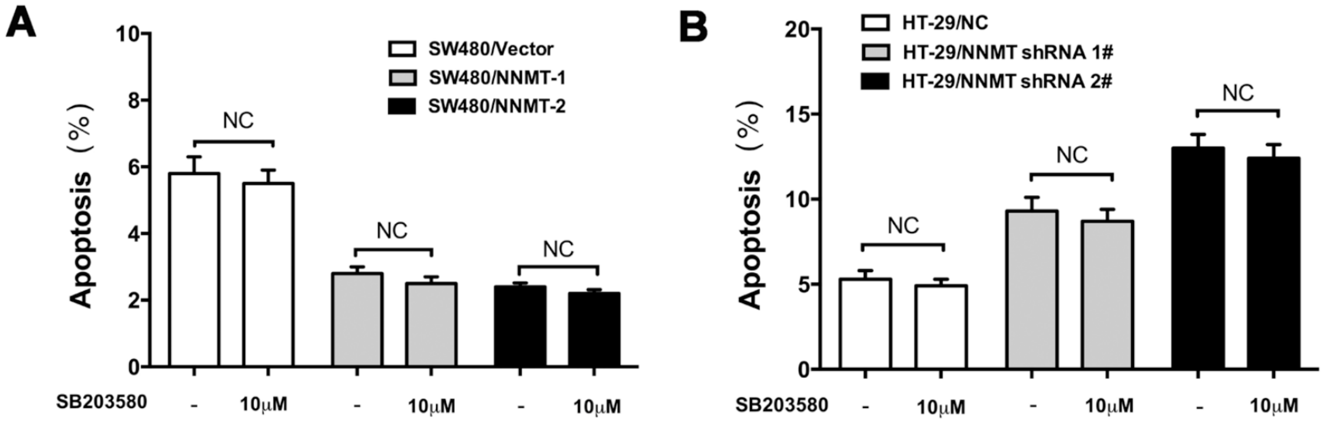

Supplementary Figure S3: The effect of SB203580 on the apoptosis in SW480 and HT-29 cells. Cells were treated with SB203580 (10μM) for 48 h. Apoptosis was evaluated by Annexin V-PE and 7-AAD. **A, B.** Histogram shows the combination of the results of three independent experiments. Data are presented as Mean ± SD (n = 3).
